# Could Cyclosiversioside F Serve as a Dietary Supplement to Prevent Obesity and Relevant Disorders?

**DOI:** 10.3390/ijms241813762

**Published:** 2023-09-06

**Authors:** Siqi Qin, Junren Chen, Kexin Zhong, Dan Li, Cheng Peng

**Affiliations:** State Key Laboratory of Southwestern Chinese Medicine Resources, School of Pharmacy, Chengdu University of Traditional Chinese Medicine, Chengdu 611137, China

**Keywords:** Cyclosiversioside F, obesity, obesity-relevant disorders, diabetes mellitus, mechanisms of action

## Abstract

Obesity is the basis of numerous metabolic diseases and has become a major public health issue due to its rapidly increasing prevalence. Nevertheless, current obesity therapeutic strategies are not sufficiently effective, so there is an urgent need to develop novel anti-obesity agents. Naturally occurring saponins with outstanding bio-activities have been considered promising drug leads and templates for human diseases. Cyclosiversioside F (CSF) is a paramount multi-functional saponin separated from the roots of the food-medicinal herb Astragali Radix, which possesses a broad spectrum of bioactivities, including lowering blood lipid and glucose, alleviating insulin resistance, relieving adipocytes inflammation, and anti-apoptosis. Recently, the therapeutic potential of CSF in obesity and relevant disorders has been gradually explored and has become a hot research topic. This review highlights the role of CSF in treating obesity and obesity-induced complications, such as diabetes mellitus, diabetic nephropathy, cardiovascular and cerebrovascular diseases, and non-alcoholic fatty liver disease. Remarkably, the underlying molecular mechanisms associated with CSF in disease therapy have been partially elucidated, especially PI3K/Akt, NF-κB, MAPK, apoptotic pathway, TGF-β, NLRP3, Nrf-2, and AMPK, with the aim of promoting the development of CSF as a functional food and providing references for its clinical application in obesity-related disorders therapy.

## 1. Introduction

Obesity is a metabolic syndrome caused by a complex interaction between environmental and genetic factors, which mainly include, but are not limited to, imbalanced diet, endocrine dyscrasia, genetic and epigenetic regulation, sociodemographic influences, as well as intrauterine condition [1,2]. The incidence of obesity is increasing rapidly worldwide, and without appropriate management, the global prevalence will hit 18% in men and above 20% in women by 2025 [3]. One of the most critical phenotypes of metabolic abnormalities in obesity is identified as adipose tissue dysfunction [4]. In an obese state, adipose tissue fails to store excessive energy and may give rise to fat deposits in other tissues concerned with metabolic homeostasis, such as skeletal muscle and liver. Such ectopic fat deposits could further cause inflammatory reactions, insulin resistance, dysregulation of adipokine secretion, and disruption of mitochondrial function [5]. Chronic low-grade inflammation in adipose tissue has been considered a dominating risk factor for obesity and metabolic complications [6], which is triggered by dysfunctional adipocytes and infiltration of bone marrow-derived immune cells [7]. In addition, insulin signaling pathways, involving a network of tightly connected cascades, also account for an essential role in the obesity progression [8]. Nevertheless, impairment of insulin signaling can trigger metabolic imbalances that result in obesity and related diseases like type 2 Diabetes Mellitus (T2DM) [9]. Simultaneously, the phosphorylation of insulin receptors becomes less sensitive to insulin during insulin resistance, which further affects glucose uptake, glycogen synthesis, as well as gluconeogenesis [10].

It is well-established that obesity underlies various debilitating disorders covering diabetes mellitus, cardiovascular diseases, hypertension, hyperlipidemia, and non-alcoholic fatty liver disease [11,12]. Actually, obesity elevates glucose intolerance as well as insulin resistance (IR) and aggravates hyperglycemia and dyslipidemia, which makes the management of DM become more intractable [5,13]. Long-term exposure to high glucose (HG) causes neurological, microvascular, and macrovascular damage and low immune response in the body, which subsequently gives rise to the occurrence of complications, such as peripheral neuropathy, nephropathy, and refractory wound [14]. Furthermore, obese individuals are more susceptible to cardiovascular diseases like hypertension; 78% of men with essential hypertension and 65% of women with essential hypertension are reported to be caused by excessive weight gain [15]. Remarkably, the activation of the renin-angiotensin-aldosterone system and sympathetic nervous system, as well as the physical pressure of the kidneys induced by visceral fat, are important mechanisms of obesity-induced hypertension [16]. Further, the increasing incidence and severity of non-alcoholic fatty liver disease (NAFLD) is strongly related to obesity, which plays an essential part in the initial process leading to simple steatosis and its progression to non-alcoholic steatohepatitis through disturbing adipokines, inflammatory cytokines, as well as adipose tissue-derived hormones [17]. However, given that the onset and development of obesity involves the regulation of multiple genetic and environmental factors, its prevention and treatment are more intractable than anticipated [18]. Although several drugs have been approved for the treatment of obesity at present, most of them are characterized by a single pathway of action and severe side effects, leading to unsatisfactory treatment results [19,20]. Therefore, it is critical to seek safe and effective drug candidates for the treatment or prevention of obesity and its complications.

**Figure 1:**
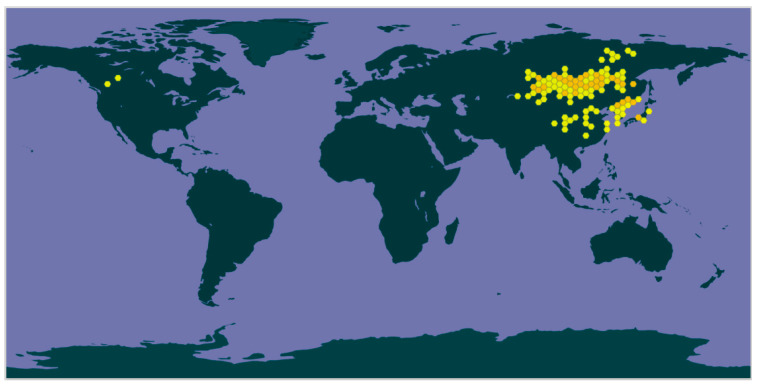
Geographical distribution of *Astragalus mongholicus* Bunge. Data from the Global Biodiversity Information Facility.

Food-origin saponins are valuable templates for the development of specific bioactive agents, which make an outstanding contribution to human health [21]. *Astragalus mongholicus* Bunge, a perennial belonging to the family Fabaceae, is native to Siberia to the Russian Far East as well as Western and Northern China and widely distributed in temperate Asia (Figure 1). So far, 3689 *Astragalus* species have been recorded according to the database of Global Biodiversity Information Facility (https://www.gbif.org (accessed on 11 April 2023)), despite the fact that a few other Astragalus species have been demonstrated to induce serious toxicity in livestock due to chemical components like indolizine alkaloids [22], these components have not been identified in *Astragalus mongholicus* in dietary supplements [23]. *Astragalus mongholicus* has been used for centuries in many East Asian countries to improve health [24,25], and it is also an integral ingredient in Lectranal, a clinically proven natural product for allergic reactions. 

**Figure 2:**
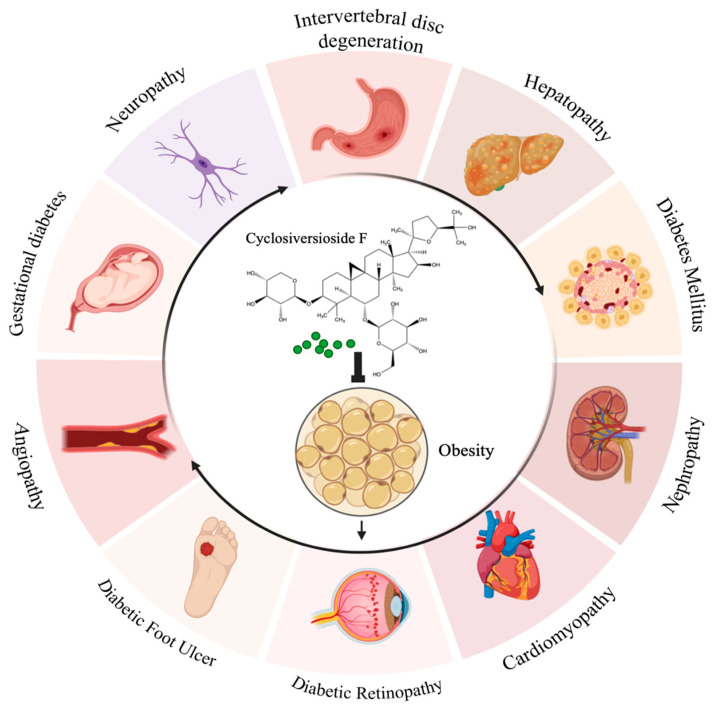
Chemical structure of Cyclosiversioside F and its potential roles in obesity and associated disorders. Cyclosiversioside F possesses promising therapeutic effects against obesity, DM, and a series of complications, including nephropathy, cardiomyopathy, diabetic retinopathy (DR), diabetic foot ulcer, angiopathy, neuropathy, intervertebral disc degeneration, hepatopathy, and gestational diabetes.

Cyclosiversioside F (CSF) is one of the most paramount ingredients separated from the functional food *Astragalus mongholicus*, which possesses various bioactivities, such as anti-inflammation [26], anti-cancer [27], anti-oxidative stress [28], and lowering lipid as well as blood glucose [29,30]. In recent years, CSF has emerged as a promising therapeutic agent for metabolic disorders, especially obesity [31], DM [32], NAFLD [33], cardiovascular and cerebrovascular diseases [34,35,36], diabetic nephropathy [37,38], and so on. Simultaneously, the potential mechanisms of CSF in regulating these diseases have been partially elucidated, which principally include interfering with PI3K/Akt-centered signals [39], NF-κB pathway [40], MAPK pathway, apoptotic pathway [41], TGF-β pathway [42], NLRP3 [43], Nrf-2 pathway [44], AMPK pathway [29], Wnt pathway [45], and integrin/IKL signal, eNOS/NO signal [46], and others. The chemical structure of CSF and its potential roles in obesity and associated disorders have been portrayed in Figure 2. 

Since CSF is derived from the edible plant *Astragalus mongholicus* and possesses favorable bioactivities, can it be developed as a functional food to fight obesity and related complications? In this systematic review, the publications over the past decades collected from PubMed, Google Scholar, and Web of Science databases were summarized. The keywords used for the search were CSF, obesity, obesity-related diseases, and mechanisms of action. In this paper, the mechanism of CSF discovered through in vitro and in animal models are presented, which are important for undertaking further research that assesses the usefulness of CSF in the prevention and/or treatment of obesity and other disorders arising on its basis.

## 2. Effects of CSF on Signals-Associated with Obesity and Associated Disorders

### 2.1. PI3K/Akt Pathway

Interfering with PI3K/Akt-centered signaling pathways could be one of the primary mechanisms of CSF in alleviating physiological damages triggered by obesity, hypertension, and other complications. CSF was shown to improve leptin resistance by down-regulating leptin level and the expression of p-PI3K, suppressor of cytokine signaling 3 (SOCS3), and protein-tyrosine phosphatase 1B (PTP1B) while up-regulating the expression of p-STAT3, leptin receptor, and pro-opiomelanocortin in high-fat diet (HFD)-induced hypertension rats [47]. Further, CSF exerted a promising protective effect on β cells under diabetic conditions, which reversed uric acid (UA)-induced Min6 cell viability reduction, suppressed apoptosis, and blocked decreased insulin secretion. Remarkably, the inhibitory effect of CSF against UA-triggered pancreatic β-cell damage was conferred through modulating the PI3K/AKT pathway, as evidenced by the up-regulation of p-Akt. Nevertheless, wortmannin, the inhibitor of the PI3K/AKT pathway, countervailed these protective effects of CSF [39]. Further, CSF was demonstrated to ameliorate oxidative stress and apoptosis in streptozotocin (STZ)-induced INS-1 cells via modulating Akt/GSK-3β/Nrf-2 signaling pathway, as reflected by the increases in Akt, Nrf-2, and GSK-3β. Likewise, the inhibitor of Akt reversed such effects of CSF in INS-1 cells [48], which revealed that targeting PI3K/AKT signaling pathway is the possible mechanism of CSF in rescuing β-cell damage during DM. In addition, CSF could modulate adipocyte inflammation induced by DM. CSF administration resulted in the up-regulation of C1q tumor necrosis factor-related protein 3 (CTRP3), which accelerated PI3K/AKT pathway activation in 3T3-L1 cells, thereby alleviating IR and inflammatory response, as evidenced by the elevated expression of GLUT-4, CTRP3, p-PI3K, as well as p-AKT and the decreased levels of IL-6 and TNF-α. Interestingly, the protective effect of CSF on 3T3-L1 cells was inhibited by CTRP3 siRNA and Wortmannin [49]. CSF promoted the intake of glucose in insulin-treated C2C12 myotubes by activating the IRS1/AKT pathway, as evidenced by the increase in p-IRS1, AKT, and GLUT4 [50]. Furthermore, a recent study found that CSF administration obviously relieved metabolic dysfunctions, decreased fasting blood glucose (FBG), triglyceride (TG), and low-density lipoprotein (LDL), and elevated high-density lipoprotein (HDL) in T2DM mice. CSF exerted its hypoglycemic effects in T2DM mice and IR-induced HepG2 cells by activating the PI3K/AKT signal [51]. Taken together, these findings illustrated that CSF might attenuate β-cell dysfunction, adipocyte inflammation, insulin resistance, and impaired glucose intake triggered by DM via interfering with pathways connected with PI3K/Akt. 

Additionally, CSF also displayed promising therapeutic effects on diabetic nephropathy (DN) via an Akt-signal mediated axis. Treatment of CSF caused a significant inhibitory effect on cell growth and ROS generation in HG-induced MCs, which markedly reduces NADPH oxidase activity and the protein expression level of Nox4. In addition, CSF application significantly down-regulated HG-induced Akt phosphorylation in the MCs and enhanced Ca^2+^ influx, suggesting that CSF protected MCs from HG-induced damage by suppressing NADPH oxidase/ROS/Akt pathway [52]. Further, CSF protects human glomerular endothelial cells (GEnCs) from HG and high insulin invasion by improving the integrity of the cell filtration barrier by increasing the expressions of ZO-1 and claudin-5. In addition, CSF administration suppressed inflammation and oxidative stress via increasing SOD and decreasing TNF-α, IL-1β, and MDA. Moreover, CSF down-regulated the expression of p-AKT and up-regulated that of p-GSK3α [53], which suggested that the therapeutic effect of CSF on DN might be relevant to its capability to modulate AKT/GSK signaling pathway. Furthermore, CSF intervention relieved polydipsia, polyuria, and albuminuria in DN mice and alleviated the injury of glomerular as well as tubular via reducing glomerular tuft area (GTA), glomerular tuft volume (GTV), glomerular basement membrane (GBM), N-acetyl-β-d-glucosamine-dase (NAG), neutrophil gelatinase-associated lipocalin (NGAL), and TGF-β1. Noticeably, CSF down-regulated the levels of p-Akt and p-mTOR [54], which suggested that CSF could ameliorate the renal injury induced by DM via suppressing the Akt/mTOR signaling pathway. 

Through modulating the PI3K/Akt signaling pathway, CSF might be potentially useful in alleviating diabetic vascular complications, diabetic retinopathy, diabetic peripheral neuropathy (DPN), diabetic cardiomyopathy, as well as gestational diabetes. CSF intervention mitigated apoptosis in ox-LDL-treated HUVECs via up-regulating the expression of miR-140-3p and down-regulating the expression of p-PI3K and p-Akt. Intriguingly, inhibition of miR-140-3p expression or overexpression of KLF4, a valid target of miR-140-3p, countervailed these effects of CSF [55], which implied that CSF might relieve endothelial damage induced by DM via regulating miR-140-3p/KLF4 axis-dependent PI3K/Akt pathway. Further, CSF is capable of relieving the apoptosis of retinal pigment epithelial cells in STZ-induced diabetic rats via modulating the miR-128-mediated PI3K/AKT/Fas signaling pathway. CSF intervention down-regulated the levels of Fas, caspase-9, HOXB3, p-PI3K, p-AKT, and p-p70S6K1, while increased FasL and miR-128 both in diabetic rats and HG-treated RPE cells [56]. Likewise, Zhao et al. observed that CSF alleviated the disorder appearance of new vessels, the thinning of retinas, and the increase in blood glucose in STZ-induced diabetic retinopathy (DR) rats via up-regulating the expressions of p-AKT, p-PI3K, AKT1, and CTNNB1 [57]. Moreover, CSF administration significantly alleviated the increased blood glucose levels in HFD-induced DPN rats and improved sensory nerve conduction velocity (SCV), motor nerve conduction velocity (MCV), as well as myelin lesions. Remarkably, CSF increased the expression of miR-155 in HG-induced RSC96 cells and HFD-induced DPN rats, which suppressed PI3K/Akt/mTOR pathway activation, thereby contributing to cellular autophagy [58]. Thus, it suggested that regulating miR-155/PI3K/Akt/mTOR might be a possible mechanism of CSF in DPN therapy. In addition, CSF could relieve diabetic cardiomyopathy via regulating autophagy and myocardial lipid metabolism, preventing apoptosis, alleviating myocardial inflammation, and improving myocardial energy metabolism. Mechanically, CSF administration reduced autophagosomes, lowered the ratio of LC3II/LC3I, increased Bcl-2 and p-AKT expression, decreased miR-34a expression in HG-induced H9C2 cells [36], which suggested that targeting AKT/Bcl2/(LC3II/LC3I) axis might be a possible way of CSF in diabetic cardiomyopathy therapy. It is remarkable that CSF could ameliorate genetic mice with gestational diabetes by promoting the phosphorylation of Akt, reducing cAMP accumulation, and decreasing hepatic gluconeogenesis [59], suggesting that CSF could be a potential drug candidate for gestational diabetes by activating Akt/PDE4B pathway. 

### 2.2. MAPK Pathway

It was found that CSF might exert kidney-protective properties under hyperglycemic conditions by modulating the MAPK signaling pathway. CSF intervention inhibited HG-induced apoptosis of renal tubular epithelial cells, down-regulated the expression of TGF-β1 and p-p38, while up-regulated the levels of hepatocyte growth factor (HGF) [60]. In addition, CSF intervention significantly diminished urinary albumin excretion, alleviated kidney tubular lesions, down-regulated serum creatinine (Scr), NGAL, and NAG expression, and up-regulated HDL-C expression in STZ-induced diabetic mice by blocking the phosphorylation of MEK1/2, ERK1/2, and RSK2 [54,61]. Further, CSF ameliorated iatrogenic hyperinsulinemia-induced kidney injury in STZ-induced diabetic rats by inhibiting oxidative stress and inflammation via targeting the ERK1/2 signaling pathway. CSF intervention alleviated the proliferation of the mesangial cell, the thickening of the basement membrane, and the effacement of the podocyte foot process, decreased the levels of Col-IV, LN, Nox4, as well as ERK1/2, and increased the expression of TRPC6 [62]. Furthermore, CSF was reported to alleviate epithelial-to-mesenchymal transition (EMT) and improve DN in *db/db* mice by blocking the activation of CX3CL1-RAF/MEK/ERK pathways, which down-regulated the expression of vimentin, α-SMA, IL-1β, p-c-Raf/c-Raf, and IL18, while up-regulated the expression of E-cadherin. Notably, CX3CL1-siRNA exhibited similar effects as CSF, whereas pReceiver-M90-CX3CL1 countervailed the protective effect of CSF on EMT [63], revealing that targeting CX3CL1-mediated RAF/MEK/ERK axis might be a promising mechanism of CSF to alleviate DN. In addition, pre-treatment with CSF prevented DN deterioration in STZ-induced diabetic rats and apoptosis of tunicamycin-induced human podocytes, down-regulated CHOP and caspase-1 expression, promoted nephroprotection by inhibiting endoplasmic reticulum (ER) stress, as evidenced by the suppressed phosphorylation of PERK, eIF2α, and JNK as well as the decreased levels of GRP78 and ORP150 [64]. Similarly, CSF administration reduced apoptotic cell number in the kidneys of *db/db* and STZ-induced diabetic mice by down-regulating Podocin, Nephrin, ATF6/PERK downstream signal CHOP, IRE1α downstream signals p-JNK as well as cleaved caspase-12 levels, and rescued SERCA activity. Furthermore, the expression of Bcl-2, SERCA2, and GPR78 was enhanced in the renal cortex of *db/db* mice after CSF intervention, while that of Bax, cytochrome c, APAF1, and AIF was down-regulated. Moreover, CSF application suppressed ER stress and restored basal cytosolic Ca^2+^ levels by inhibiting PTP proteins [65,66]. Interestingly, CSF administration obviously relieved vacuolation of renal tubular epithelial cells and glomerular fibrosis lesions, suppressed apoptosis in HG-treated NRK-52E cells, and alleviated metabolic dysfunctions in HFD-induced DN KKAy mice via interfering with H3K4me1/MAP4K3 axis, as evidenced by the down-regulation of MAP4K3 expression as well as the inhibited binding of H3K4me1 and MAP4K3 [67]. In brief, regulating MEK1/2, ERK1/2, JNK, and MAP4K3 signal cascades might underlie the effects of CSF in mitigating various pathological processes during DN. 

Moreover, a recent study found that targeting the JNK/Nrf-2 pathway might be one of the crucial mechanisms of CSF in treating diabetic ketoacidosis. CSF administration alleviated oxidative stress in STZ and alloxan-induced DKA mice via promoting p-JNK, Nrf-2 activity, as well as related antioxidant enzymes SOD, CAT, and GSH-Px expression, and down-regulating MDA levels. Furthermore, the JNK pathway activator (Anisomycin) exhibited the equivalent effect as CSF, while the JNK pathway inhibitor (SP600125) inhibited the antioxidant effect of CSF [68], suggesting that CSF could function as a candidate drug for diabetic ketoacidosis. In addition, suppressing the activation of JNK signaling might be a possible mechanism of CSF in mitigating diabetic angiopathy. It was demonstrated that CSF intervention improved the viability of HG-treated HUVECs by down-regulating the expression of IL-1β, TNF-α, JNK, and p-ASK1 [69]. Further, targeting P2X7R/p38 cross-talk has been recognized as a promising strategy of CSF in improving diabetic endothelial dysfunction. Leng et al. reported that CSF suppressed the activation of p38 induced by P2X7R, which resulted in ameliorated endothelial dysfunction in STZ-induced diabetic rats and HG-induced RAOEC cells, as reflected by the alleviation of oxidative stress and inflammatory responses. Remarkably, the p38 inhibitor SB203580p38 MAPK and P2X7R siRNA displayed the equivalent efficacy of CSF [41]. Furthermore, CSF treatment decreased the percentage of apoptotic cells in H9C2 cells triggered by HG and high fat, down-regulated p-p38 and p-JNK levels, and enhanced p-ERK amount, which were similar to the effects induced by MAPK inhibitors [70], suggesting that regulating MAPK pathway might be a pivotal mechanism of CSF in preventing diabetic cardiovascular disease. Noticeably, targeting ERK1/2- NF-κB axis might be a promising way for CSF in the treatment of DR. Ding et al. demonstrated that CSF intervention relieved metabolic dysfunctions in *db/db* mice with DR via blocking ERK1/2 phosphorylation and NF-κB pathway activation, which also suppressed AR activity, reduced the numbers of apoptosis of retinal RGCs, and down-regulated IL-1, IL-6, VEGF, VCAM-1, TNF-α, as well as MMPs expression [71]. Taken together, these findings elucidated that the protective effects of CSF on ketoacidosis, angiopathy, myocardial infarction, and retinopathy triggered by DM might be partially ascribed to its capacity to modulate MAPK signal as well as the cross-talks covering JNK/Nrf-2, P2X7R/p38, and ERK1/2-NF-κB. 

### 2.3. NF-κB Pathway

Interfering with NF-κB and relevant cross-talks might be another pivotal mechanism of CSF in ameliorating atherosclerosis, hepatic steatosis, hypertension, DM, and its complications. CSF alleviated the atherosclerosis and hepatic steatosis in HFD-induced LDLR^−/−^ mice by suppressing the lipid deposition and inflammation via inactivating MAPK/NF-κB signaling pathway, which reduced the levels of IL-1β, IL-6, TNF-α, iNOS, and VCAM-1, and down-regulated the expression of p65, JNK, ERK1/2, and p38. Further, CSF exerted a protective effect in the liver and aortic sinus, as evidenced by the increase in collagen deposition and the expression of α-SMA, the decrease in caspase 3, TG, and total cholesterol (TC) expressions, and the reduction in aspartate aminotransferase (AST) and alanine aminotransferase (ALT) levels [72]. Moreover, CSF was illustrated to alleviate NAFLD in rats via restraining the TLR4/NF-κB signaling pathway. CSF treatment improved the hepatic steatosis, intralobular inflammation, and balloon-like changes while decreasing the lipid deposition in HFD rats by decreasing the levels of TG, AST, and ALT and down-regulating the expression of TLR4, MyD88, NF-κB, TNF-α, IL-6, as well as IL-8 [73]. In addition, CSF alleviated obesity-induced hypertension in HFD rats via suppressing NF-κB-mediated inflammatory response. CSF application decreased p-IKKβ, NF-κB, IL-1β, and TNF-α in the hypothalamus and adipose tissue via augmenting the expression of α7nAChR, while these effects were countervailed by α7nAChR blocker [47]. Further, Li et al. found that CSF displayed promising therapeutic effects on diabetic hyperglycemia, which reversed HG-induced MSCs cell viability reduction and enhanced cell proliferation via blocking NF-κB pathway activation. CSF application down-regulated the expression of TLR4 and NF-κB p65 while elevated the expression of MMP-2. Remarkably, CSF diminished the expression of MCP-1 and TNF-α in the serum of diabetic patients and attenuated the inflammatory response [40]. 

Further, CSF was found to mitigate DN by targeting NF-κB-mediated inflammation, which ameliorated podocyte foot process effacement and albuminuria, alleviated the injury of glomerular and tubular, as well as extracellular matrix accumulation in STZ-induced diabetic rats through decreasing the expression of NF-κB, p65, a1-chain type IV collagen, MCP-1, ICAM-1, and TNF-α [54,74]. Likewise, in HFD-induced DN mice, CSF improved renal dysfunction, decreased urine albumin creatinine ratio (ACR) levels, ameliorated thylakoid proliferation and expansion, enhanced autophagy, down-regulated α-SMA, FN, and Col IV levels. In addition, CSF intervention promoted the activation of MCs through restraining p65 acetylation, decreasing of mAlb, and increasing Beclin 1 and LC3 II via enhancing SIRT1 levels [75]. CSF treatment down-regulated HG-induced phosphorylation and degradation level of IκBα, alleviated the reduction in TRPC6, and enhanced Ca^2+^ influx in the MCs [52]. Furthermore, CSF ameliorated HG-triggered podocytes EMT in mouse podocyte cell line and polygenic KK-Ay mice by enhancing autophagy via targeting the SIRT-NF-κB p65 pathway. Specially, CSF treatment decreased FN, Col IV, and p-65, increased the SIRT1 in KK-Ay mice, up-regulated the E-cadherin, nephrin, SIRT1, Beclin I, and LC3 II, and down-regulated the TGF-β, α-SMA, N-cadherin, and p65 in mouse podocyte cell line. Interestingly, the inhibitor of SIRT1 and NF-κB p65 and 3-Methyladenine (3-MA) reversed the protective effect of CSF [76], which revealed that CSF might protect DN by promoting autophagy via targeting SIRT-NF-κB p65 pathway. 

CSF administration obviously relieved the increased blood glucose levels and the body weight loss, improved the structure of the aortic endothelium wall, and down-regulated FPG and HbAlc in HFD, HG, and STZ-induced diabetic rats. In addition, CSF improved endothelial dysfunction via enhancing the release of NO and eNOS and blocking the activation of MCP-1, TNF-α, and p65 [77]. Further, CSF ameliorated dysfunction in HUVECs caused by HG, which decreased ICAM-1, VCAM-1, TLR4, and p65, as well as down-regulated IL-6 and TNF-α. Notably, the effects of TLR4 and p65 inhibitors on HUVECs were similar to that of CSF, whereas the inhibitor of NO synthesis reversed the protective effects of CSF [78]. Further, CSF intervention relieved body weight loss and metabolic dysfunctions in *db/db* mice with DR. The protective effect of CSF on RGC dysfunction in DR mice was found to be implicated in the blockade of ERK1/2 phosphorylation and NF-κB pathway activation. CSF treatment suppressed AR activity, reduced the numbers of apoptosis of retinal RGCs, and down-regulated IL-1, IL-6, VEGF, VCAM-1, TNF-α, as well as MMPs expression [71], which suggested that targeting ERK1/2-NF-κB might be a promising axis for the treatment of DR. Additionally, CSF was reported to attenuate palmitate-induced inflammation in insulin-treated C2C12 myotubes by decreasing MCP-1, IL-6, TNF-α, and TLR4 via inactivating the IKK/IκBα pathway, indicating that CSF might be a potential candidate agent for diabetic cardiopathy [50]. CSF alleviated gestational diabetes exacerbation and metabolic dysfunctions in mice by suppressing the TLR4/NF-κB pathway, as evidenced by the decrease in TLR4 and p-p65 [79]. Taken together, this evidence implied that targeting NF-κB and relevant cross-talk, including TLR4, SIRT1, and ERK1/2, might be the possible mechanism of CSF in relieving DM, DN, DR, diabetic cardiovascular diseases, and gestational diabetes. 

### 2.4. Apoptotic Pathway

CSF might be a therapeutic agent for DN via inhibiting the ER-stress-induced cell apoptosis. CSF treatment ameliorated epithelial cells exfoliation, dilation of tubular, basement membrane of renal tubules thickening, collagen deposition, and the apoptosis of renal tubular epithelial cells in diabetic rats and apoptosis ratio in HG-treated podocyte through down-regulating the expressions of Bax, caspase-12, cytochrome c, BIP, APAF1, AIF, caspase-9, and caspase-3, while up-regulating Bcl-2, and suppressed the ER-stress by diminishing the expressions of GRP78, p-eIF2α, p-PERK, TRB3, ATF4, ATF6, p-IRE1α, XBP1, and CHOP, while up-regulating SERCA2b [66,80,81,82]. Notably, both SERCA2b silencing and the inhibitors of the autophagy pathway abolished the protective effects of CSF on HG-treated podocyte cells [65]. Thus, these phenomena suggested that CSF could ameliorate apoptosis via down-regulating the PERK-ATF4-CHOP and SERCA2 pathways. In addition, CSF protected podocytes from apoptosis triggered by DN via targeting TRPC6 mediated the calcineurin/NFAT signaling pathway. CSF intervention alleviated the HG-triggered mouse podocytes apoptosis via reversing the increase in TRPC6, restoring the level of Ca^2+^, and suppressing the nuclear translocation of NFAT2, but TRPC6 siRNA partly inhibited these effects of CSF [82,83]. Intriguingly, CSF restored basal cytosolic Ca^2+^ levels by inhibiting PTP proteins [66]. Taken together, the anti-apoptotic effect of CSF in DN might be relevant to its capability to regulate Ca^2+^ homeostasis in podocytes. Moreover, Chen et al. demonstrated that CSF could effectively alleviate palmitic acid (PA)-induced HK-2 cell injury, which protected renal tubular epithelial cells from apoptosis by restraining ROS production, up-regulating Bcl-2, and down-regulating Bax, Cleaved Caspase-9 as well as Cleaved-caspase3 expression [44,84]. Additionally, similar phenomena could be observed in CSF-treated diabetic rats, accompanied by the improved thickening of mesangial, ameliorated effacement of podocyte foot process, and relieved albuminuria [85]. Furthermore, CSF ameliorated podocyte apoptosis by targeting the lncRNA-TUG1/TRAF5 signaling pathway in STZ-induced diabetic rats. CSF intervention up-regulated the levels of lncRNA-TUG1 as well as TUG1 and down-regulated TRAF5 in HG-induced mouse podocytes and diabetic rats, whereas both lncRNA-TUG1 knockdown and si-TUG1 abolished the decrease in TRAF5 triggered by CSF as well as the protective effect of CSF on podocytes apoptosis in rats [86]. Collectively, these findings revealed that targeting the lncRNA-TUG1/TRAF5 signaling pathway might be a new mechanism of CSF in attenuating DN.

CSF was reported to improve intervertebral disc degeneration induced by DM via an apoptosis-mediated pathway, which ameliorated the morphology and viability of HG-treated nucleus pulposus cells (NPCs), attenuated senescence and apoptosis through improving the telomere length and activation of telomerase, as evidence by the down-regulated expressions of cleaved-caspase 3, Bax, FITC, and p-16 and up-regulation of TERT and Bcl-2 [87]. In addition, CSF could protect against myocardial injury induced by T2DM via regulating the mitochondrial apoptotic pathway. CSF treatment relieved myocardial fibers damage and myocardial hypertrophy in diabetic rats, down-regulated the protein expressions of ANP, BNP, Cyt C, and cleaved caspase-3 in heart tissues and HG-treated H9C2 cells, and improved myocardial energy metabolism via up-regulating the ratio of ATP/ADP and ATP/AMP as well as the protein levels of PGC-1α and NRF1 [88]. Further, targeting the mitochondrial apoptotic pathway was found to be a possible mechanism of CSF in treating DPN as well. Ben et al. demonstrated that CSF reduced pain tolerance, accelerated nerve conduction velocity, and increased MNCV and SNCV in STZ-induced diabetic rats by mitigating mitochondrial damage and restoring mitochondrial function via modulating the SIRT1/p53 pathway, as evidenced by the elevation of SIRT1, and the depression of p53, Drp1, and Bax/Bcl-2 [89]. 

### 2.5. TGF-β Pathway

Targeting the TGF-β signaling pathway was illustrated to be a promising mechanism of CSF in attenuating the deterioration of DN. CSF intervention significantly diminished HG-induced EMT and arrested HG-induced NRK-52E cell viability and apoptosis via blocking the activation of TGF-β1 and Smad pathways, as reflected by the down-regulation of TGF-β1, α-SMA, HG, p-Smad2, N-cadherin, and vimentin, and up-regulation of E-cadherin and ocludin. In addition, the protective effect of CSF on EMT was suppressed by the TGF-β1 activator SRI-011381 hydrochloride [42]. Further, CSF ameliorated HG-triggered podocytes EMT in mouse podocyte cell line and polygenic KK-Ay mice through decreasing FN, Col IV, TGF-β, α-SMA, and N-cadherin, while up-regulating E-cadherin and nephrin [76]. Su et al. reported that CSF delayed the progression of DN via down-regulation of CD36 expression, which alleviated PA-induced fibrosis and oxidative stress in HMCs, diminished lipid retention, prevented activation of TGF-β1/Smad2/3 pathway, and decreased the expression of FN, Col4A1, TGF-β1, p-Smad2/3, ROS, NOX4, as well as p22phox. Interestingly, CD36 inhibitor sulfo-N-succinimidyl oleate exhibited similar effects to CSF [90]. Moreover, the renal-protective effect of CSF under diabetic conditions might be conferred by targeting the miRNA-mediated TGF-β1/Smad axis. Mao et al. found that CSF treatment down-regulated the expression of miR-192 in HG-induced RMCs and STZ-induced DN rats, which resulted in the suppression of the TGF-β1/Smad pathway [91]. Furthermore, CSF was illustrated to up-regulate Smad7 and nephrin while down-regulate miR-21, α-SMA, β-catenin, TGF-β1, and P-Smad3 in primary podocytes, MCs, as well as HFD-induced DKD mice. Further, CSF suppressed the fibrosis of the kidney and improved renal function and morphology by decreasing the levels of ACR, mAlb, Col IV, and FN. Notably, the inhibitors of Wnt/β-catenin pathway and TGF-β1/Smads pathway altered the effect of CSF in miR-21-overexpressed podocytes and MCs [92], which suggested that inhibiting the overexpression of miR-21-induced podocyte dedifferentiation and MC activation might be a possible mechanism of CSF in relieving renal fibrosis induced by DM. 

Additionally, CSF could promote diabetic wound healing via elevating cell proliferation, re-epithelialization, and angiogenesis via the TGF-β/Smad pathway. Gao et al. demonstrated that CSF accelerated wound healing via up-regulating TGF-β1, p-Samad2, and p-Samad3 and down-regulating Samd7 in HG-induced HaCaT cells. In addition, CSF relieved oxidative stress and inflammatory responses through increasing SOD, IL-10, and TGF-β1 while down-regulating ROS, MDA, IL-6, as well as IL-8 [93]. Furthermore, CSF was shown to reverse myocardial fibrosis in T2DM rats and HFD rats by reducing the level of the TGF-β1 and alleviating myocardial inflammation via down-regulating the expression of TNF-α, IL-6, and IL-1β, accompanied by the increase in left ventricular systolic pressure, a decrease in left ventricular end-diastolic pressure, and elevation of TC, TG, HDL, as well as free fatty acid (FFA) [35], which suggested that the protective effects of CSF on diabetic cardiomyopathy might be partially ascribed to the regulation of TGF-β signal. 

### 2.6. NLRP3

CSF treatment significantly decreased TG urinary albumin, the total protein of urinary, albumin-to-creatinine ratio, creatinine clearance rate, mean glomerular volume, and fibrotic area in the glomeruli in *db*/*db* mice. Furthermore, CSF partly reversed reductions of podocin and synaptopodin, improved NLRP3 inflammasome-mediated inflammation by down-regulating NLRP3, pro-caspase-1, caspase-1, as well as IL-1β in the renal cortex and serum of *db/db* mice, and restored the viability of podocytes induced by HG [37]. Further, CSF was demonstrated to alleviate ox-LDL-induced endothelial progenitor cell (EPC) dysfunction by enhancing cell viability, proliferation, migration, and tube formation. Notably, CSF and the inhibitor of LOX-1 attenuated oxidative stress and inflammation by down-regulating ROS, IL-1β, IL-6, IL-10, TNF-α, LOX-1, NLRP3, ASC, and caspase1, whereas the overexpression of LOX-1 reversed the inhibition of CSF, indicating that CSF protects EPCs from ox-LDL via suppressing the LOX-1/NLRP3 signaling pathway [94]. Therefore, targeting the mediated LOX-1/NLRP3 pathway might be a new strategy for vascular diabetic complications. Moreover, Zhang et al. found that CSF administration significantly alleviated weight loss, enhanced insulin levels, diminished blood glucose levels in C57BL/KsJ-Lepdb/+ mice, and ameliorated the reduced fetal number as well as elevated body weight in the offspring mice. In addition, CSF attenuated the oxidative stress and inflammatory response in C57BL/KsJ-Lepdb/+ mice via decreasing the number of inflammatory cells, downregulating TNF-α, MDA, IL-1β, Casp-1 p10, and IL-6, promoting the expression of related antioxidant enzymes such as SOD, GSH, CAT, and GPx. In addition, CSF might function as an inhibitor of the inflammasome, which rescued the increase in NLRP3 in C57BL/KsJ-Lepdb/+ mice and suppressed inflammasome assembly [43]. Altogether, targeting the NLRP3 inflammasome-mediated signaling pathway was considered a possible mechanism of CSF in ameliorating DN, diabetic vascular diseases, as well as gestational diabetes. 

### 2.7. Nrf-2 Pathway

CSF was shown to relieve β cell damage induced by DM via targeting cross-talk between Nrf-2 and Akt signals. Lin et al. found that CSF application reversed the decrease in Nrf-2 and p-Akt in STA-treated INS-1 cells, which was accompanied by the down-regulation of Bax, caspase-3, and MDA as well as elevation of Bcl-2, SOD, and GSH-Px [48]. In addition, CSF could effectively alleviate PA-induced HK-2 cell injury via regulating Nrf-2-mediated oxidative stress, which restrained ROS production, up-regulated Bcl-2 and p-Nrf-2, and down-regulated Bax and cleaved-caspase3 expression, thereby alleviating the apoptosis of HK-2 cell [84]. Interestingly, by interfering with the Nrf-2/ARE/L-FABP signaling pathway, CSF might also participate in the improvement in DN. Wang et al. reported that CSF intervention mitigated oxidative stress in HG-treated HK-2 cells through enhancing Nrf-2 and its target genes HO-1, Keap1, and NQO1, and restraining the activity of LPO, ROS, as well as L-FABP [44]. Additionally, targeting the miR-138-mediated Nrf-2 signaling pathway might be a possible mechanism of CSF in alleviating ferroptosis during DR. Tang et al. observed that CSF improved the survival rates of ARPE-19 cells under HG conditions, ameliorated mitochondrial morphology, and degraded the peroxidation of lipid and oxidative stress. In addition, CSF treatment up-regulated the levels of Sirt1, GPX4, GCLM, GCLC, and Nrf-2 in nuclear and down-regulated the expression of ROS and miR-138-5p. Noticeably, the inhibitor of Sirt1 and miR-138-5p overexpression decreased the levels of Nrf-2 in nuclear and Sirt1, and suppressed the protective effect of CSF in ARPE-19 cells [95], which suggested that CSF inhibited the ferroptosis through up-regulating the Sirt1/Nrf-2 signaling pathway via down-regulating the miR-138-5p. Moreover, CSF displayed therapeutic effect on neuropathy triggered by DM, which ameliorated cognitive impairment in diabetic mice through a Nrf-2-dependent cascade. CSF treatment alleviated metabolic dysfunction, promoted cognitive performance, and mitigated neuronal damage in T2DM mice, accompanied by the down-regulation of ROS, MDA, IL-1β, IL-6, as well as TNF-α and the elevation of SOD. Additionally, the neuroprotective effect of CSF in T2DM mice may be associated with up-regulation of Nrf-2, HO-1, NQO1 and down-regulation of Keap1 [96]. Thus, it suggests that targeting Nrf-2/Keap1/HO-1/NQO1 might be a promising axis for CSF in alleviating neuroinflammation and oxidative stress during DM-related cognitive impairment. 

### 2.8. AMPK Pathway

Modulating the AMPK signaling pathway was regarded as a promising mechanism of CSF in alleviating NAFLD and DM. Wang et al. demonstrated that CSF might function as an AMPK activator that prevented lipid accumulation in IR HepG2 cells. Specifically, CSF intervention suppressed the intracellular accumulation of glucose, down-regulated the expression of TG and FFA, curbed the nuclear transcription of SREBP-1c by activating AMPK, down-regulated the levels of SREBP-1c and its downstream factors FAS as well as SCD, up-regulated the activity of AMPKα1, AMPKα2, as well as IRS-2, and restored the levels of ACC1 and SREBP-2c, accompanied by the restoration of lipid metabolism. Notably, the APMK inhibitor compound C can eliminate the effects of CSF [97]. Likewise, CSF inhibited FFA-induced ER stress and lipid accumulation via activating the AMPK signal. CSF administration promoted phosphorylation of AMPK, SREBP-1c, as well as ACC, downregulated SREBP-1 expression, and reduced ACC1, FAS, SCD1, GRP78, CHOP, and p-PERK. Simultaneously, the AMPK activator AICAR exhibited an effect comparable with CSF, while these effects induced by CSF were abolished by the AMPK inhibitor [29], which suggested that CSF might be a possible agent for hepatic steatosis therapy via targeting AMPK-dependent signal cascades. 

Additionally, CSF administration obviously relieved the metabolic dysfunctions, enhanced the mRNA expression of AMPK and SIRT1, and up-regulated the protein expression of p-AMPK and SIRT1 in T2DM mice. Notably, similar results could be observed in IR HepG2 cells [51], which suggested that CSF exerted hypoglycemic effects both in vivo and in vitro via activating AMPK/SIRT1. Further, CSF ameliorated the liver injury in HFD and STZ-induced diabetic rats, which facilitated the homeostasis of glucose, dyslipidemia, as well as the deposition of liver lipid, increased HO-1, and decreased TNF-α and IL-6. Notably, CSF accelerated autophagy via up-regulating the Beclin1, LC3I, and p-AMPK while down-regulating P62 and p-mTOR [32]. In addition, CSF might function as a candidate agent for DN by regulating AMPKα-regulated autophagy. CSF alleviated DN progression by suppressing the hypertrophy of the glomerular, the expansion of the mesangial matrix, and glomerulosclerosis, down-regulating the activity of the renin-angiotensin system (RAS), improving albuminuria, podocyte apoptosis, and inflammation in STZ-induced diabetic mice. Further, CSF facilitated autophagy via increasing LC3A/B, Beclin-1, Atg12, and p-AMPKα and decreasing p62, p-mTOR, as well as p-p70S6K. Indeed, the inhibitors of the autophagy pathway and AMPKα abolished the protective effects of CSF on HG-treated podocyte cells [65]. 

### 2.9. Wnt Pathway

CSF treatment reduced renal tubular epithelial cell vacuolization, decreased the levels of Scr and blood urea nitrogen (BUN) in DN rats and improved the viability of HG-induced HK-2 cells by increasing SOD and down-regulating ROS, MDA, IL-1, IL-4, as well as IL-6. Moreover, CSF postponed EMT advancement via blocking the activation of the Wnt/β-catenin pathway, as evidenced by the elevation of APC, Axin, GSK-3β, and E-cadherin and the diminution of N-cadherin, α-SMA, Wnt1, β-catenin, Vimentin, FN, as well as Col-IV [98]. In addition, Zeng et al. revealed that CSF intervention significantly diminished urinary albumin excretion, decreased urinary NAG, and rescued ALB levels in puromycin aminonucleoside (PAN)-treated rats, which were accompanied by the elevation of podocin, nephrin, as well as synaptopodin. Interestingly, CSF prevented podocyte cytoskeleton form PAN-triggered damage in rats and human podocytes via activating the Wnt/PCP pathway, as evidenced by the up-regulation of Wnt5a, PTK7, ROCK1, and p-JNK [45]. Thus, targeting the Wnt/PCP pathway might be a promising mechanism of CSF in protecting the podocyte cytoskeleton.

### 2.10. Integrin/ILK Signal

CSF pre-treatment significantly decelerated the excretion of proteinuria in STZ-induced DN rats in a dose-dependent manner. Simultaneously, thylakoid expansion, glomerulosclerosis, and interstitial fibrosis were alleviated in STZ-induced DN rats after CSF intervention. Further, CSF prevented the shedding of podocyte foot process and reduced podocyte density in DN rats by up-regulating integrin α3 and integrin β1 expression, down-regulating β1-ILK expression, and reducing the number of WT-1 positive cells [99]. Additionally, CSF intervention accelerated the proliferation of glomerular mesangial cells and mesangial matrix and promoted the formation of thicker basement membranes in renal tissue of DN rats by accelerating the expression of podocytes adhesion molecules and sustaining the stability of podocyte cytoskeletal proteins, down-regulating ILK and α-actinin-4 expression, and the up-regulating β1 integrin expression [38]. Taken together, these findings manifested that reducing podocyte detachment and promoting cell adhesion via modulating integrin/ILK signal might be one of the crucial mechanisms of CSF in relieving DN. 

### 2.11. eNOS/NO Signal

CSF repaired vasoconstriction damage, enhanced NO bioavailability, elevated p1177eNOS content, and modified endothelial dysfunction in STZ-induced diabetic mice. In addition, the oxidative stress is alleviated after CSF intervention, accompanied by the decrease in NADPH-related oxidase, down-regulated expressions of SOD, MDA, p22phox, p47phox, p67phox, NOX2, NOX4, as well as Rac-1, and up-regulated levels of SOD [100]. Further, CSF alleviated the acetylcholine-mediated endothelium-dependent relaxation and oxidative stress caused by hyperglycemia both in STZ-induced diabetic rats and HG-induced HUVECs. CSF down-regulated the levels of calpain-1 and ROS while up-regulated the SOD, GSH-Px, eNOS, and NO, suggesting that CSF might ameliorate diabetic endothelial dysfunction via inactivating calpain-1 and enhancing the eNOS/NO signaling [101]. Moreover, CSF was considered as a potential agent for DN via modulating the levels of NO and eNOS. CSF alleviated renal injury by suppressing the expansion of the mesangial matrix, decreasing the apoptosis of kidney cells, and reducing albuminuria, BUN, Scr, and HbA1c in STZ-induced DN rats. Further, in HG-treated human renal glomerular endothelial cells, CSF decreased the cells’ apoptosis and permeability and improved the proliferation of cells. Notably, CSF up-regulated the levels of NO and phosphor-Ser1177 eNOS while inhibiting the acetylation of eNOS [46]. Collectively, these findings reveal that blocking oxidative stress, increasing NO bioavailability, and suppressing acetylation of eNOS might be responsible for the protective effect of CSF on diabetic endothelial dysfunction and DN.

### 2.12. Free Radicals

Considerable evidence elucidated that scavenging free radicals might be a crucial mechanism of CSF in ameliorating NAFLD, DN, DR, and diabetic hepatopathy. CSF intervention significantly rescued weight and liver weight gains in HFD-induced NAFLD mice, which restored liver damage through downregulating 5-LO, LTA4H, as well as LTB4. CSF administration was reported to alleviate oxidative stress in PA-induced LO2 cells, accompanied by the reduction in lipid accumulation, increased expression of GSH-Px, and decreased expression of TG, TC, ROS, and MDA [33]. CSF attenuated EMT in NRK-52E cells triggered by glycated albumin (GA) through inhibiting oxidative stress, which reduced intracellular ROS accumulation and NADPH oxidase, decreased the mRNA expression of α-SMA, and improved the mRNA expression of E-cadherin [102]. In addition, CSF was demonstrated to ameliorate oxidative stress in retinal capillary endothelial cells (RCECs) triggered by HG. CSF treatment down-regulated the expression of GLUT1 and increased the levels of SOD, CAT, and GSH-PX while decreasing the levels of MDA, ROS, GSSG, and Nox4, which revealed that CSF prevented RCECs from oxidative stress by balancing the levels of ROS production and the enzymes of antioxidant [103]. CSF was also considered a potential drug candidate for the prevention of diabetic hepatopathy. Han et al. reported that CSF intervention obviously relieved the body weight loss and metabolic dysfunctions in high-lipid and HG-induced DM rats, which modulated blood lipids, alleviated liver lesions, and decreased FBG, TG, TC, ALT, AST, as well as ALP. In addition, after CSF application, apoptosis and oxidative stress in diabetic rats were mitigated, which was accompanied by the elevation of SOD, GSH-Px, as well as CAT and the down-regulation of MDA [104]. 

### 2.13. HIF-1α/VEGF

Coordinating the activation of the HIF-1α/VEGF signaling pathway might be responsible for the molecular basis of CSF in promoting diabetic wound healing. CSF intervention accelerated the closure of the wounds in diabetic mice by elevating the number of alternative activated macrophages F4/80+CD206+ cells, up-regulating the expression of fibronectin, collagen IIIa, VEGF, vWF, arginase-1, Ym1, as well as IL-13, and down-regulating the activities of SOD, CAT, and TNF-α. Further, topical application of CSF improved the formation of thick granulation tissue with more new blood vessels, stimulated re-epithelialization, and promoted collagen synthesis and deposition in diabetic wounds [105]. In addition, WANG et al. found that CSF up-regulated the levels of SUMO1, SAE1, SAE2, Ubc9, SENP1, PCNA, Ras, HIF-1α, PPARγ, and VEGFR2 in HG-treated HUVECs [106], which indicated that the therapeutic effects of CSF on diabetic wound healing might be conferred through activating SUMO/HIF-1α Pathway. Thus, it suggested that CSF might be a candidate agent for diabetic ulcers. 

## 3. Toxicity of Cyclosiversioside F

Exploring the side effects or risks associated with CSF is of great significance for the development of CSF as a dietary supplement. So far, potential maternal toxicity and fetal toxicity of CSF have been investigated in rats and rabbits [107]. CSF was shown to possess maternal toxicity in rats at the dose of 1.0 mg/kg after intravenous administration and display fetal toxicity in rats and rabbits at a dose higher than 0.5 mg/kg. However, CSF did not exhibit adverse effects on skeletal development in fetuses of rats and rabbits and induced any classical teratologic changes at the test doses [108]. In addition, it was reported that a significant delay in reaction time of the fur development, eye-opening locomotor activity, and cliff parry reflex could be found in pups after maternal rats exposed to CSF at the dose of 1.0 mg/kg for continuous 4 weeks, whereas no deficiency in memory and learning of F1 pups was discovered [109]. Taken together, these findings illustrated that women need to be more cautious if they want to use CSF to treat obesity and relevant diseases during pregnancy. Nonetheless, the current evidence on whether CSF causes any toxicity in humans is insufficient and needs to be supplemented by more preclinical and clinical studies.

## 4. Conclusions and Perspective

At present, remarkable progress has been made in the investigation of the pathophysiological mechanisms underlying the occurrence and development of obesity. Growing studies reported that obesity appears to be the consequence of disturbed brain circuits and impaired feedback of neuroendocrine, which is connected to pathological overeating as well as lack of exercise [1]. More importantly, obesity is descendible and predisposed to a variety of disorders, which implies that some obese individuals are affected by monogenetic mutation, and a large proportion of the obese population is likely to develop DM, NAFLD, hypertension, atherosclerosis, and other metabolic disorders. Accordingly, the treatment strategies for obesity should be multifaceted, and the development of multi-targeted drugs is a priority for future research. The discovery of novel agents to combat obesity and obesity-related diseases from the daily diet seems feasible, as these potential functional foods are safe enough to be consumed for long periods of time [110]. 

**Figure 3:**
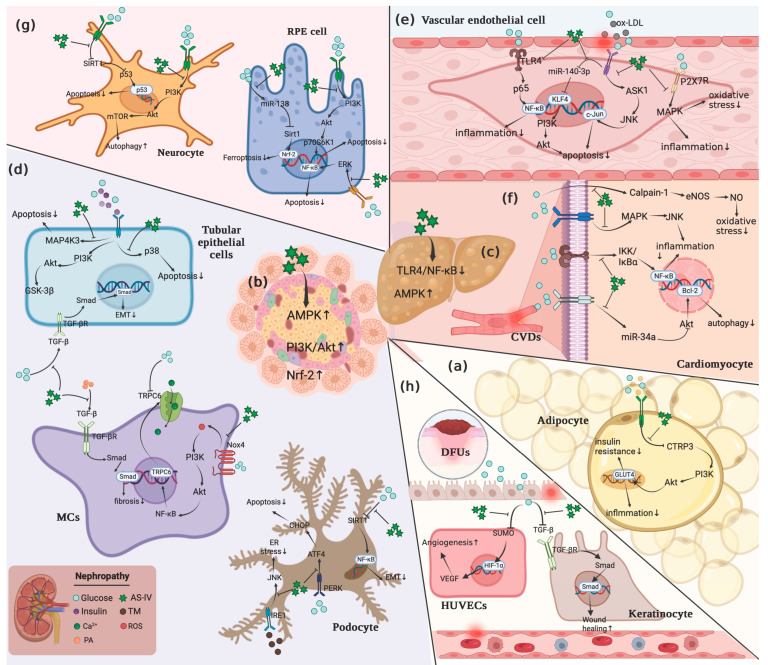
Possible mechanisms of CSF in alleviating obesity and obesity-relevant disorders. CSF suppressed adipocyte inflammation during obesity by suppressing the CTRP3/PI3K/Akt pathway (**a**), alleviated DM through activating AMPK, PI3K/Akt, and Nrf-2 pathways (**b**), and relieved NAFLD by inhibiting TLR4/NF-κB pathway and activating AMPK pathway (**c**). In addition, CSF mitigated DN via interfering with PI3K/Akt, MAPK, NF-κB, apoptotic pathway, TGF-β, Nrf-2, and AMPK (**d**), prevented obesity-related CVDs by modulating PI3K/Akt, MAPK, NF-κB, TGF-β, eNos/NO signals (**e**,**f**), ameliorated DR by regulating MAPK, NF-κB, and Nrf-2 pathways (**g**), attenuated obesity-induced neuropathy via mediating PI3K/Akt, apoptosis, and Nrf-2 (**g**). Moreover, CSF improved diabetic wound healing through targeting HIF-1α/VEGF and TGF-β/Smad signals (**h**).

CSF, derived from the root of the edible herb *Astragalus mongholicus* Bunge, has been demonstrated to possess a variety of bioactivities, such as attenuating adipose tissue inflammation, relieving oxidative stress, reducing blood glucose, regulating lipid metabolism, protecting β cell damage, and improving insulin resistance, etc. Based on these outstanding bioactivities, CSF displays promising therapeutic effects on obesity and relevant diseases. So far, considerable studies have been devoted to elucidating the potential mechanisms of CSF in the prevention or treatment of these diseases, which, although not comprehensive, could advance the development of CSF as an anti-obesity drug candidate. From these studies, it could be inferred that interfering with PI3K/Akt-centered signaling cascades, MAPK, NF-κB, and apoptotic pathways might be the most critical mechanism of CSF in treating obesity, hypertension, NAFLD, DM, obesity-induced cardiovascular and cerebrovascular diseases, retinopathy, neuropathy, as well as gestational diabetes. In addition, CSF also modulated signals, including TGF-β, NLRP3, Nrf-2, AMPK, Wnt, integrin/ILK, eNOS/NO, and free radicals to exert its protective effects against obesity-derived diseases. Interestingly, there existed several cross-talks between these signaling pathways, like MAPK-NF-κB and ERK1/2-NF-κB, which might explain, in part, the therapeutic effects of CSF in different metabolic diseases at similar dosages. The primary underlying mechanisms of CSF in alleviating obesity and related disorders have been portrayed in Figure 3. However, the current evidence is mostly from preclinical studies with more in vitro trials, and future studies should focus on in vivo trials as well as clinical trials to facilitate the possibility of developing CSF as a functional food against obesity and associated disorders. 

## Abbreviations

ALT	alanine aminotransferase
AST	Aspartate aminotransferase
BUN	blood urea nitrogen
CSF	Cyclosiversioside F
CTRP3	C1q tumor necrosis factor-related protein 3
DM	Diabetes Mellitus
DN	Diabetic Nephropathy
DPN	Diabetic Peripheral Neuropathy
DR	Diabetic Retinopathy
EMT	epithelial-to-mesenchymal transition
EPCs	endothelial progenitor cells
ER	endoplasmic reticulum
FBG	Fasting Blood Glucose
FFA	Free Fatty Acid
GA	glycated albumin
GBM	glomerular basement membrane
GSK-3β	glycogen synthase kinase-3β
GTA	glomerular tuft area
GTV	glomerular tuft volume
HDL	High Density Lipoprotein
HFD	High Fat Diet
HG	High glucose
HGF	hepatocyte growth factor
ILK	integrin/integrin-linked kinase
IR	Insulin resistance
LDL	Low Density Lipoprotein
MCV	motor nerve conduction velocity
NAFLD	Non-Alcoholic Fatty Liver Disease
NAG	N-acetyl-β-d-glucosamine-dase
NGAL	neutrophil gelatinase-associated lipocalin
NPCs	nucleus pulposus cells
PA	Palmitic acid
PAN	puromycin aminonucleoside
PTP1B	protein-tyrosine phosphatase 1B
RAS	renin-angiotensin system
Scr	serum creatinine
SCV	sensory nerve conduction velocity
SOCS3	Suppressor of cytokine signaling 3
STAT3	signal transducer and activator of transcription-3
STZ	streptozotocin
T2DM	Type 2 Diabetes Mellitus
TC	total cholesterol
TG	triglyceride
UA	uric acid
